# Paeoniflorin Inhibits ASK1-TF Axis by Up-Regulating SOCS3 to Alleviate Radiation Enteritis

**DOI:** 10.3389/fphar.2022.743708

**Published:** 2022-03-14

**Authors:** Lei Sheng, Fan Hu, Hanqing Yu, Xueyou Tao, Rumeng Jia, Yufeng Gu, Lu Chen, Hong Kong, Chen Miao, Wenjing Fei, Yang Yang, Jinhui Jia, Xia Zhu, Xueming He, Liang Hu, Jianxin Ma, Wen-Tao Liu, Mi Yang

**Affiliations:** ^1^ The Comprehensive Cancer Centre of Nanjing Drum Tower Hospital, Clinical College of Nanjing Medical University, Nanjing, China; ^2^ Jiangsu Key Laboratory of Neurodegeneration, Department of Pharmacology, Nanjing Medical University, Nanjing, China; ^3^ Department of clinical laboratory, Children’s Hospital of Nanjing Medical University, Nanjing, China; ^4^ Department of Anesthesiology, Yangzhou Maternal and Child Health Hospital Affiliated with Yangzhou Medical University, Yangzhou, China; ^5^ The Affiliated Cancer Hospital of Nanjing Medical University and Jiangsu Cancer Hospital and Jiangsu Institute of Cancer Research, Nanjing, China; ^6^ Department of Orthopedics, Jiangsu Province Hospital of Integration of Chinese and Western Medicine, Nanjing, China; ^7^ Center for Clinical Research and Translational Medicine, The Affiliated Lianyungang Oriental Hospital of Kangda College of Nanjing Medical University, Lianyungang, China; ^8^ The Comprehensive Cancer Centre of Nanjing Drum Tower Hospital, The Affiliated Hospital of Nanjing University Medical School, Nanjing, China

**Keywords:** radiation enteritis, paeoniflorin, SOCS3, ASK1, TF

## Abstract

Radiation enteritis is one of the main adverse effects of radiotherapy, presenting with a poorly understood etiology and limited options for therapy. Intestinal inflammation and ischemia are the core mechanisms of radiation enteritis. Suppressor of cytokine signaling 3 (SOCS3) is an endogenous “inflammation brake.” We hypothesized that paeoniflorin, a pinane monoterpene bitter glycoside, could increase SOCS3 expression to reduce inflammation and ischemia and improve enteritis in mice. Laser Doppler flowmetry was used to detect changes in intestinal blood flow. RAW264.7 and human umbilical vein endothelial cells were used to investigate the mechanism of action of paeoniflorin. It was observed that radiation caused high mortality, intestinal inflammatory responses, and low blood flow in mice. Paeoniflorin effectively alleviated intestinal atrophy, prevented thrombosis, improved radiation enteritis, and reduced mortality in mice undergoing radiotherapy. In addition, paeoniflorin increased the release of growth arrest-specific gene 6 (Gas6) and phosphorylation of the Axl receptor, subsequently inducing the expression of SOCS3 and inhibiting the expression of p-apoptosis signal-regulating kinase 1 and tissue factor *in vivo* and *in vitro*. Based on our findings, we suggest that paeoniflorin is potentially effective in alleviating radiation enteritis *via* the activation of the Gas6/Axl/SOCS3 axis and subsequent reduction in intestinal inflammation and ischemia.

## Introduction

Radiation enteritis (RE) is a gastrointestinal complication caused by radiotherapy that affects up to 13% of patients (Modlin et al., 2005). The primary clinical manifestations are nausea, vomiting, abdominal pain, diarrhea, and tenesmus. Currently, most clinical treatment methods are limited to the alleviation of symptoms, such as diarrhea and bloody stools, caused by enteritis, including antibacterial and anti-infective drugs. Unfortunately, the efficacy of these treatments is far from satisfactory owing to the condition’s complex molecular mechanisms.

Intestinal inflammation is involved in the development of chemotherapy-induced enteritis. Extensive research has focused on the production of inflammatory cytokines (Gérard et al., 2006), destruction of intestinal immune and chemical barriers, intestinal flora disorders (Guipaud et al., 2018), and changes in cell structure (Abayomi et al., 2009). Recently, increasing evidence has shown that damage to the microvascular endothelium of the intestine is also involved (Waddell et al., 1999; Zimmerer et al., 2008). Wang et al. (2004) demonstrated that the expression of tissue factor (TF) and thrombin receptor increased in the intestinal vascular endothelial cells of rats after radiotherapy, and intervention with hirudin and thrombin inhibitors showed a significant protective effect against intestinal radiation toxicity. TF is rarely present in blood circulation or in contact with circulating blood; indeed, it is exposed to the peripheral circulating blood only when the integrity of the vessel wall is compromised, in order to activate the cascade of blood coagulation (Herkert et al., 2002). Thus, high expression of TF may induce microthrombus formation, ischemia, and disorder of the intestinal microcirculation system, which may be an important pathological mechanism for the persistence of RE.

Radiation therapy leads to the production of reactive oxygen species and endogenous ligands such as alarmins, which further activate Toll-like receptor 4 (TLR4), and, subsequently, the apoptosis signaling kinase 1 (ASK1)/NF-κB-based inflammatory pathway (Wang et al., 2019). Inhibition of the MAPK pathway can reduce the expression of TF induced by lipopolysaccharide (LPS) in monocytes and endothelial cells (Chen et al., 2020). In addition, activation of the transcription factor NF-κB is necessary for maximizing TF expression (Oeth et al., 1994; Mackman, 1996). As the upstream core factor of MAPK, ASK1 plays an important role in upregulating the expression and activity of TF (Mackman et al., 1991; Chu et al., 2001). Therefore, inhibiting the TLR-4/ASK-1/TF signaling pathway may be an effective way to alleviate RE. Considering the serious liver toxicity of TLR-4 inhibitors, such as eritoran (E5564) and TAK242, there is an urgent need to find a safe and effective drug or a new treatment method to inhibit TLR4 signaling.

TAM receptors (TYRO3, Axl, and Mer) belong to the receptor tyrosine kinase family. In the inflammatory self-regulation cycle, the activation of Axl signaling blocks the pro-inflammatory pathway, inducing transcription of anti-inflammatory inhibitors of cytokine signaling protein 3 (SOCS3) to inhibit the TLR signaling pathway (Rothlin et al., 2007). TAM receptor activation is inseparable from the binding of the growth arrest-specific gene 6 (Gas6). It has been reported that overexpression of Gas6 could inhibit the production of proinflammatory factors in the synovium, and immunohistochemical results have shown that the SOCS3 protein has a corresponding increasing trend (van den Brand et al., 2013). Paeoniflorin (PF) is a monoterpenoid glucoside, which has been widely used for its anti-inflammatory, hypoglycemic, and cognitive-enhancing properties, as well as other clinical medicinal values (Zhang and Wei, 2020). Our previous studies have confirmed that PF could significantly promote the expression of SOCS3 *in vivo* or *in vitro*, inhibit ASK1 expression, reduce neuroinflammation, and relieve neuropathic pain (Frobøse et al., 2006). Thus, we speculated that PF may upregulate the Axl/SOCS3 axis to reduce inflammation and TF expression to alleviate RE.

Here, we provide the first evidence that PF could alleviate RE by activating the Axl/SOCS3 axis to reduce intestinal inflammation and ischemia.

## Materials and Methods

### Animals and Treatments

Establishment of the RE model: Healthy male C57BL/6 mice weighing 18–25 g (4–6 weeks of age) were anesthetized by intraperitoneal (i.p.) injection of pentobarbital (50 mg/kg). The mice received a single irradiation in the entire abdomen under the 6-MV electron line. The irradiation range was from the xiphoid to the pubic symphysis, the distance from the skin was 10 cm, the dose rate was 260–300 MU/min, and the total amount of radiation was 14 Gy. After irradiation, the mice were returned to the animal room for normal feeding.

### Histological Analysis

On days 0, 2, 4, and 6, the colon of mice in each group was removed, and the colon tissues were completely separated and photographed. The filling degree of the colon, intestinal contents, and the thickness of the intestinal wall were observed with the naked eye to determine the degree of intestinal injury. At the same time, the length of the colon was measured. Samples were fixed in 4% paraformaldehyde at 4°C overnight, transferred to sucrose (0.3 g ml^−1^), and stored at 4°C. The samples were then embedded using conventional techniques. Sections of 5 μm were cut and stained with hematoxylin and eosin (H&E) to observe for changes in the villi, goblet cells, and crypt of intestinal mucosa.

### Cell Culture

RAW264.7 cells (ATCC number: TIB-71) were maintained in Dulbecco’s modified Eagle’s medium (DMEM, Gibco) supplemented with 10% (*v*/*v*) fetal bovine serum (FBS, Gibco) under humidified 5% CO_2_ at 37°C. Human umbilical vein endothelial cells (HUVEC) (KeyGen, KG419) were maintained in EBM-2 medium (Lonza) supplemented with SingleQuot Bullet Kit (Lonza) under humidified 5% CO_2_ at 37°C. For the LPS model, the cells were inoculated in a six-well plate and treated with different concentrations of PF 24 h later (10^−4^, 10^−5^, 10^−6^, and 10^−7^ M), 4 h before stimulation with LPS (Sigma, 1 μg/ml), and 12 h after stimulation with LPS, the protein of cells was extracted from the six-well plate.

### Intestinal Blood Flow Measurement

The intestinal blood flow was measured using laser Doppler flowmetry. Specifically, a computer-controlled optical scanner was used to direct a low-power laser beam over the exposed intestine. The mice were placed on a thermostatic table, an incision of approximately 1 cm was cut at the midline of the abdominal cavity, and a segment of the mesentery of the colon was selected and placed on a gel containing normal saline. Concurrently, the scanner head was positioned parallel to the exposed intestine at a distance of approximately 20 cm. Subsequently, a color-coded image that denoted the specific relative perfusion level was displayed on the video monitor. Blood flow values were recorded and measured using the Moor FLPIR V40 program (Gene and I Scientific. Ltd.).

### Western Blotting

Samples were collected and lysed using lysis buffer supplemented with protease inhibitors. Proteins were fractionated by SDS-PAGE and transferred to polyvinylidene fluoride membranes. Subsequently, the membranes were soaked in blocking buffer for 2 h at room temperature. After blocking, the cells were incubated overnight at 4°C with antibodies against TF (Santa Cruz Biotechnology), SOCS3 (Cell Signaling Technology), p-ASK1 (Santa Cruz Biotechnology), and β-actin (Santa Cruz Biotechnology). The blots were then incubated with secondary antibodies (Cell Signaling Technology). ImageJ software (Rawak Software Inc. Germany) was used for densitometric analyses.

### Statistical Analysis

Statistical analyses were performed using Prism 6.0 (GraphPad). The unpaired Student’s *t*-test was used to analyze the differences between the two groups. Alteration of protein expression and behavioral responses were tested with one-way analysis of variance (ANOVA), and the differences in latency over time among groups were tested with two-way ANOVA, followed by Bonferroni’s *post*-*hoc* tests. Error bars in all figures represent the mean ± standard error of the mean. The number of replicates and statistical tests are reported in all figure captions.

## Results

### Radiation Induces Intestinal Inflammation and Slow Blood Flow in Mice

**FIGURE 1:**
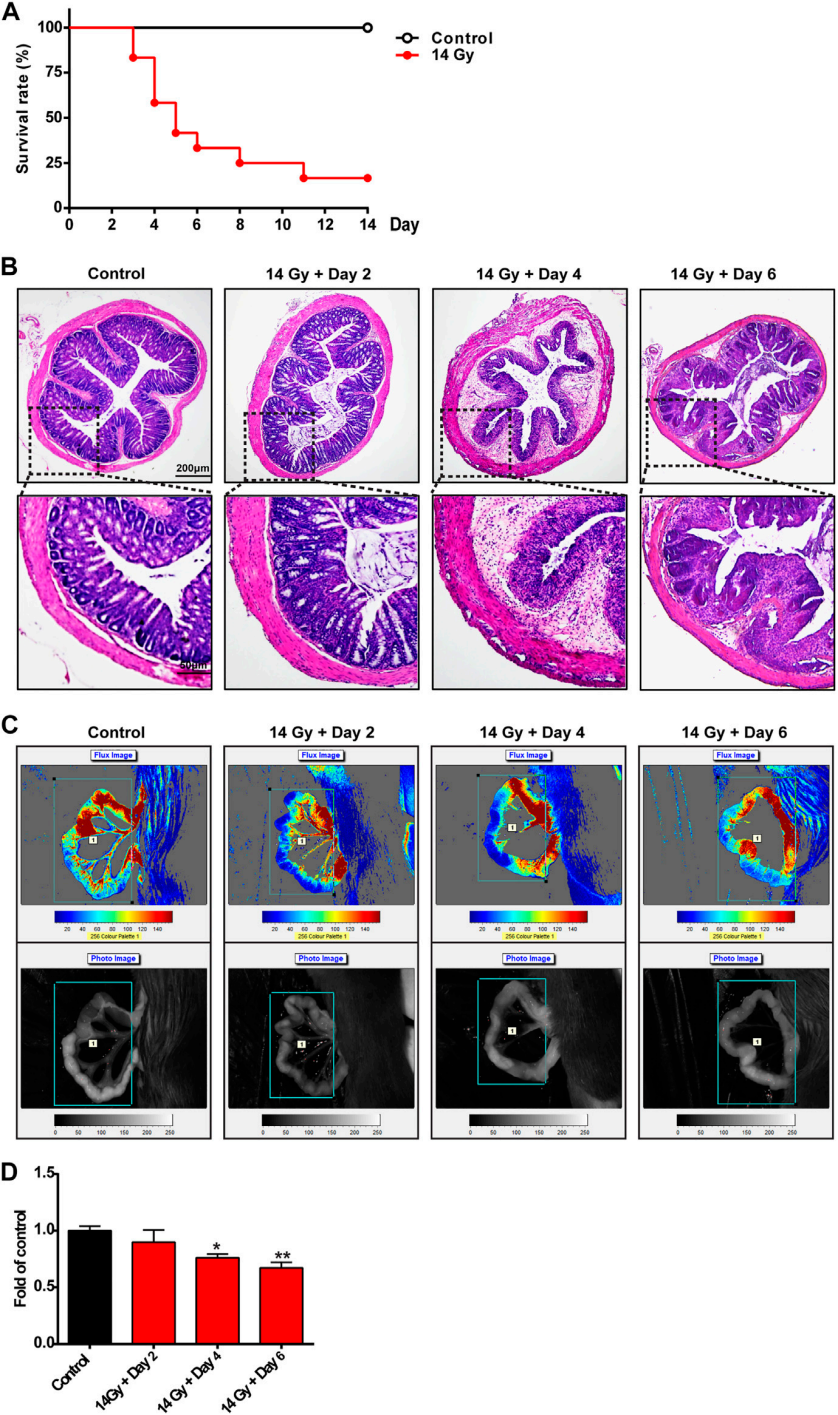
Radiation induces intestinal inflammation and slow blood flow in mice. **(A)** Survival rate of mice administered with radiation and those that were not. The implemented procedures were as follows: a single irradiation of the lower abdomen of mice with a 6-MV electronic wire; the irradiation range was from the xiphoid to pubic symphysis; the distance from the skin was 10 cm; the dose rate was 260–300 MU/min; the total amount of irradiation was 14 Gy; and the survival rate of mice was calculated during the disease process (*n* = 10 per group). Mice were exposed to 14-Gy radiation and were returned to the animal room for normal feeding during the next days. All mice were sacrificed at different time points (days 0, 2, 4, and 6). **(B)** Sections of colonic tissues were separated and stained with hematoxylin and eosin staining to observe the structure of the intestinal wall (*n* = 4 per group). The photos were observed with a confocal laser scanning microscope at ×50 and ×200 magnifications. **(C,D)** Intestinal blood flow was detected by laser Doppler and was then calculated (*n* = 5 per group). *p < 0.05 and **p < 0.01 vs. control group.

First, we investigated the survival rate, intestinal inflammation, and blood flow in mice exposed to radiation. Compared with the control group, radiation significantly decreased the survival rate of mice by as much as 30% on the 6th day (Figure 1A). As shown in Figure 1B, compared with the control group, H&E staining showed that the mucous membrane was severely damaged, the cells were arranged disorderly, and the glands became smaller and began to atrophy in the mouse colon on the 2nd day after 14-Gy irradiation. On the 6th day after irradiation, edema occurred, a large number of inflammatory cells infiltrated the colon, and the original structure of the intestine was compromised. Laser Doppler flowmetry was used to determine the blood flow of the intestinal tract of mice (Figures 1C,D); radiation markedly decreased the blood flow of the intestinal tract on the 4th and 6th day after exposure.

These data suggest that radiation could significantly increase intestinal inflammation, compromise intestinal structure, cause poor intestinal blood circulation, and decrease the survival rate of mice.

### PF Reduces Mortality and Improves RE in Mice Exposed to Radiation

**FIGURE 2:**
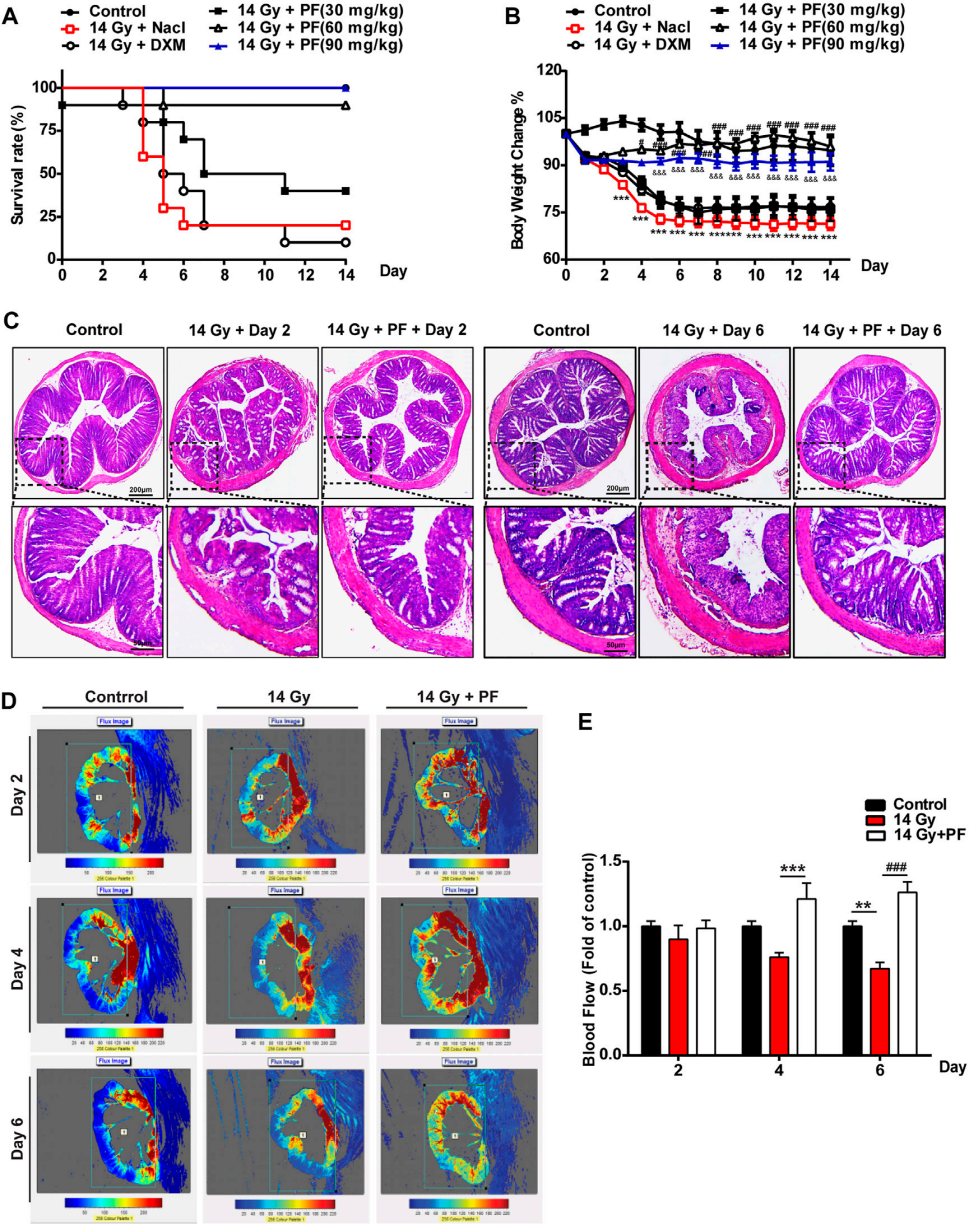
Paeoniflorin reduces mortality and improves radiation enteritis in mice exposed to radiation. Saline, dexamethasone, and different doses of paeoniflorin (PF) (30, 60, and 90 mg/kg) were injected intraperitoneally into the mice of the respective groups 2 h after they were exposed to 14-Gy radiation on the 1st day. Saline, dexamethasone, and PF were then administered every 24 h from the 2nd to the 14th day. **(A)** The survival rate of mice was calculated during the disease process (*n* = 10 per group). **(B)** The body weight changes during the disease process (*n* = 6 per group). **(C)** Sections of colonic tissues were separated and stained with hematoxylin and eosin staining (*n* = 4 per group). The photos were observed with a confocal laser scanning microscope at ×50 and ×200 magnifications. **(D,E)** The intestinal blood flow was detected by laser Doppler and was then calculated (*n* = 5 per group). **p < 0.01 and ***p < 0.001 vs. control group; ^#^ *p* < 0.05 and ^###^ *p* < 0.001 vs. 14-Gy radiation + saline group; ^&&&^ *p* < 0.001 vs. 14-Gy radiation + saline group.

To study the effect of PF on RE in mice, different doses of PF were administered intraperitoneally. As shown in Figure 2A, PF significantly decreased the mortality rate of mice exposed to radiation, especially at a dose of 90 mg/kg, which was a better outcome than that obtained with the positive control drug dexamethasone (DXM, 1.25 mg/kg, i.p.). After radiation, the appetite of the mice decreased. It was found that the weight of the mice in the radiation model group decreased compared to that of the control group, which presented a greater improvement with PF (90 mg/kg, i.p.) treatment than with DXM treatment (Figure 2B). We further measured the structure and blood flow of the colon using H&E staining and laser Doppler flowmetry. As shown in Figure 2C, compared with the radiation group on the 2nd and 6th days, PF (90 mg/kg, i.p.) decreased mucous membrane damage, recovered gland size, and reduced edema and inflammatory cell infiltration in the colon. PF (90 mg/kg, i.p.) also increased the blood flow in the colon compared to that observed in the radiation group on the 4th and 6th days (Figures 2D,E). These data suggest that PF could markedly improve RE and increase the survival rate of mice after radiation.

### PF Reduces Intestinal Injury and TF Expression *via* Inhibiting ASK1 Activity

**FIGURE 3:**
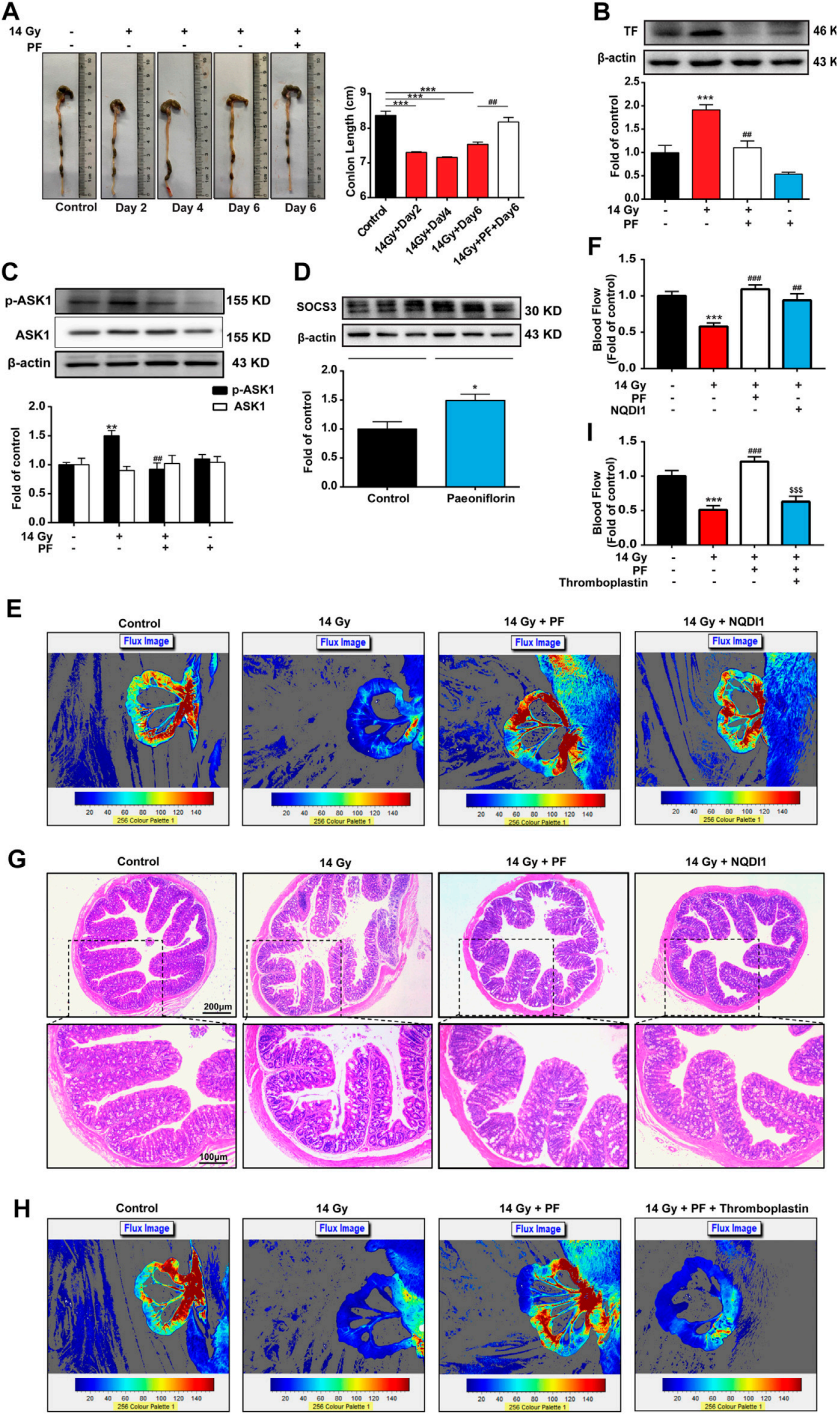
PF reduces intestinal injury and TF expression *via* inhibiting ASK1 activity. PF (90 mg/kg, intraperitoneally) was administered to radiation mice, and mice of each group were sacrificed at different time points (days 0, 2, 4, and 6). **(A)** Macroscopic images were observed, and the length of the colon from each group was measured and calculated (*n* = 4 per group). **(B–D)** Tissue factor (TF), p-apoptosis signaling kinase 1 (p-ASK1), and suppressor of cytokine signaling 3 (SOCS3) expression in the colon was determined by western blotting on the 6th day (*n* = 3 per group). **(E,F,H,I)** The intestinal blood flow was detected by laser Doppler and was then calculated on the 6th day (*n* = 5 per group). NQDI1 (4 mg/kg, i.p.) was administered 0.5 h before PF treatment. Mice were administered with thromboplastin (2.5 μg/kg, intravenously) injection within 20 s after PF treatment on the 6th day. **(G)** Sections of colonic tissues were separated and stained with hematoxylin and eosin on the 6th day (*n* = 4 per group). *p < 0.05, **p < 0.01, and ***p < 0.001 vs. control group; ^##^ *p* < 0.01 vs. 14-Gy radiation group; ^$$$^ *p* < 0.001 vs. 14-Gy radiation + PF group.

The feces, diluted feces, and water samples of the mice were easy to distinguish and observe. As shown in Figure 3A, we collected the colon of the mice and found that, in the control group, colons had black and granular contents in the intestinal tract with a normal formation of feces. Compared with the control group, the colon length was significantly shortened, and a yellow water sample appeared in the colon of mice exposed to radiation. On day 6 after radiation, PF (90 mg/kg, i.p.) clearly improved intestinal length and content status.

We further examined the expression of the coagulation factor TF related to microthrombus formation. Colon samples on day 6, administrated with or without PF, were collected to evaluate for indicators of thrombosis and further verified the effects of PF in mice. Compared with the control group, radiation increased TF expression, which was subsequently decreased by PF treatment (Figure 3B). In addition, we measured the expression of the upstream target protein, ASK1. As shown in Figure 3C, the phosphorylation of ASK1 increased after radiotherapy, whereas PF significantly reduced ASK1 phosphorylation. We also found that PF could increase the expression of SOCS3 (“inflammation brake”) in the intestinal tract of mice (Figure 3D).

Furthermore, the ASK1 inhibitor NQDI1 was used to investigate the role of ASK1 in the progression of RE. Compared with the radiation group, NQDI1 (4 mg/kg, i.p.) (Ma et al., 2020) increased the blood flow of the colon and improved intestinal ischemia in comparison to those found in the radiation group (Figures 3E, F). NQDI1 also significantly decreased mucous membrane damage, recovered gland size, and reduced edema in the colon of mice (Figure 3G).

We further verified the importance of TF in the development of RE. Thromboplastin (2.5 μg/kg, intravenously, corresponding to a dose of 2.5 μg/kg TF) (Wyseure et al., 2015) was used to set as the TF-related rescue experiments. As shown in Figures 3H,I, the protective effect of PF on intestinal blood flow was cancelled by thromboplastin.

These data suggested that PF could upregulate SOCS3 expression and inhibit the ASK1/TF axis to relieve intestinal injury.

### PF Induces SOCS3 Expression and Inhibits ASK1/TF Axis *In Vitro*

**FIGURE 4:**
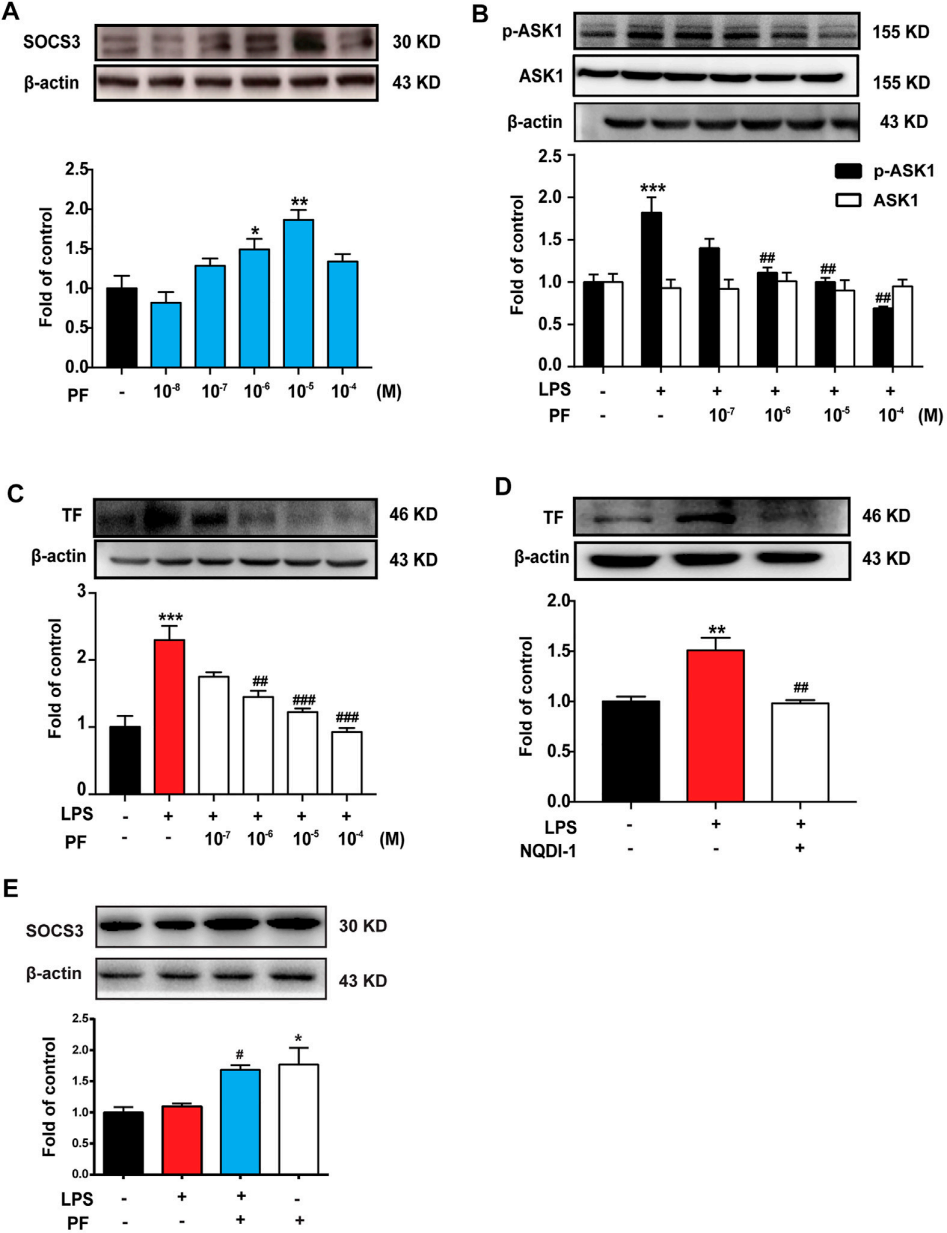
PF induces SOCS3 expression to inhibit the expression of p-ASK1 and TF in RAW264.7 cells. **(A)** RAW264.7 cells were treated with different doses of PF (10^−8^, 10^−7^, 10^−6^, 10^−5^, and 10^−4^ M) for 4 h, and the expression of SOCS3 was determined by western blotting. **(B,C)** Different doses of PF were pre-administered for 4 h, and LPS (1 μg/ml) was then administered for 12 h; the expressions of p-ASK1, ASK1, and TF were determined by western blotting. **(D)** ASK1 inhibitor NQDI-1 (600 nM) was pre-administered for 0.5 h, and LPS (1 μg/ml) was then administered for 12 h; the expression of TF was determined by western blotting. **(E)** The expression of SOCS3 after PF (10^−6^ M) administration with or without LPS (1 μg/ml). *n* = 3 per group; *p < 0.05, **p < 0.01, and ***p < 0.001 vs. control group; ^##^ *p* < 0.01 and ^###^ *p* < 0.001 vs. LPS group.

**FIGURE 5:**
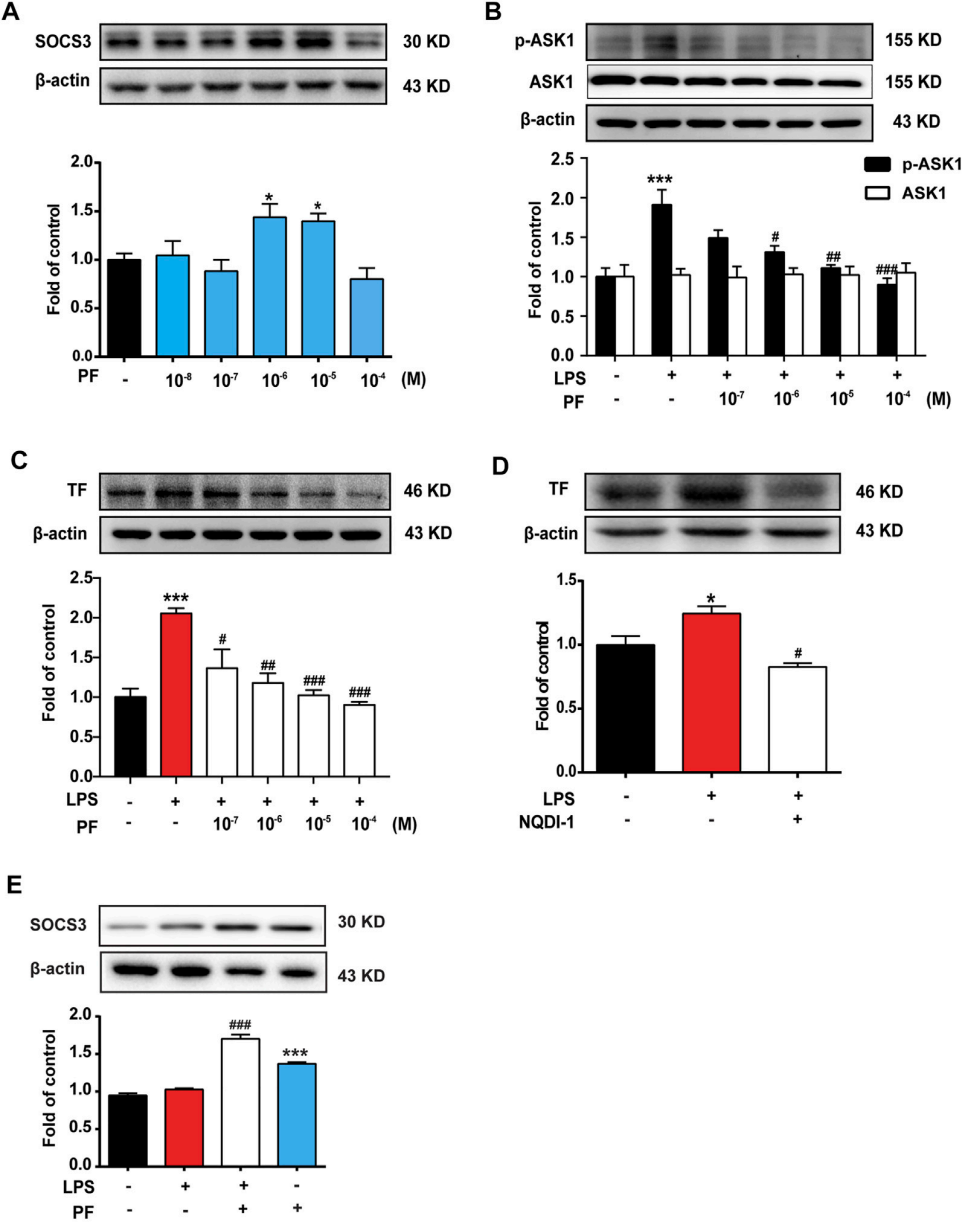
PF induces SOCS3 expression to inhibit the expression of p-ASK1 and TF in human umbilical vein endothelial cells (HUVEC). **(A)** HUVEC were treated with different doses of PF (10^−8^, 10^−7^, 10^−6^, 10^−5^, and 10^−4^ M) for 4 h; the expression of SOCS3 was determined by western blotting. **(B,C)** Different doses of PF were pre-administered for 4 h, and LPS (1 μg/ml) was then administered for 12 h; the expressions of p-ASK1, ASK1, and TF were determined by western blotting. **(D)** NQDI-1 (600 nM) was pre-administered for 0.5 h, and LPS (1 μg/ml) was then administered for 12 h; the expression of TF was determined by western blotting. **(E)** The expression of SOCS3 after PF (10^−6^ M) administration with or without LPS (1 μg/ml). *n* = 3 per group; *p < 0.05, **p < 0.01, and ***p < 0.001 vs. control group; and ^#^ *p* < 0.05, ^##^ *p* < 0.01, and ^###^ *p* < 0.001 vs. LPS group.

To explore the mechanism of PF on the expression of SOCS3, mouse monocyte macrophages (RAW264.7 cells) and HUVEC were used for *in vitro* experiments. As shown in Figures 4A, 5A, PF induced the expression of SOCS3 in RAW264.7 and HUVEC, especially at concentrations of 10^−6^ and 10^−5^ M.

RAW264.7 and HUVEC were treated with different doses of PF for 4 h and then treated with LPS (1 μg/ml) for 12 h. As shown in Figures 4B, C, 5B, C, LPS significantly increased the expression of p-ASK1 and TF in RAW264.7 and HUVEC, which were reduced by PF treatment (10^−6^, 10^−5^, and 10^−4^ M). The effect of PF on TF expression was mimicked by the ASK1 inhibitor NQDI-1 (Figures 4D, 5D). To verify the effect of PF on SOCS3 expression in the inflammatory state, data showed that PF induced the expression of SOCS3 even in the presence of LPS (Figures 4E, 5E).

These data suggested that ASK1 is an important upstream regulator of LPS-induced TF production. PF upregulates SOCS3 expression to inhibit the ASK1/TF axis.

### PF Activates Gas6/Axl Signaling to Induce SOCS3 Expression

**FIGURE 6:**
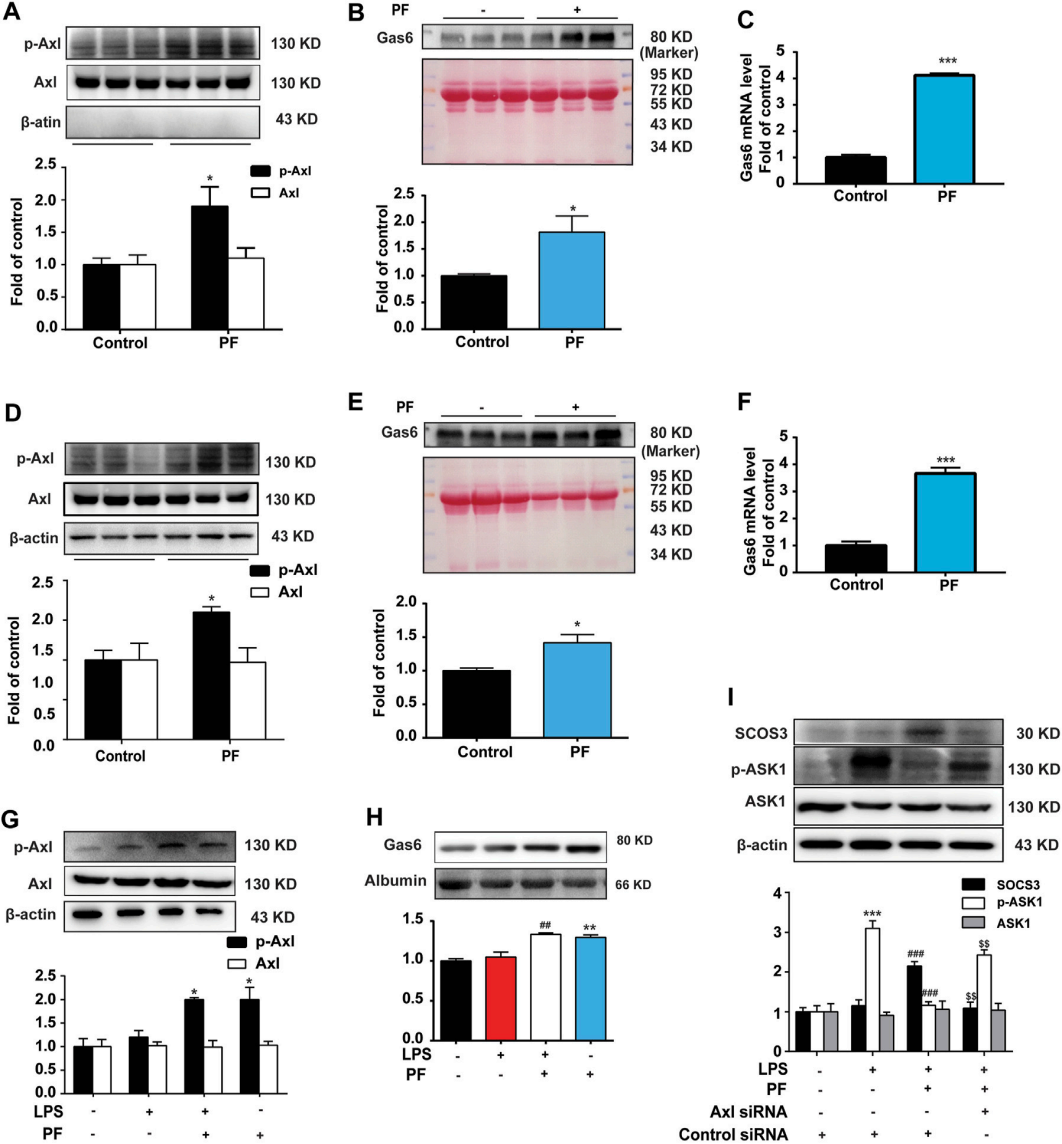
PF promotes SOCS3 expression *via* activating Gas6/Axl axis. The expression of *p*-Axl was determined by western blotting in RAW264.7 cells **(A)** and human umbilical vein endothelial cells (HUVEC) **(D)**. The level of Gas6 protein and mRNA was measured in the supernatant of RAW264.7 cells **(B,C)** and HUVEC **(E,F)**. RAW264.7 and HUVEC were administered with PF (10^−6^ M) for 4 h. The expressions of *p*-Axl **(G)** and Gas6 **(H)** after PF (10^−6^ M) administration with or without LPS (1 μg/ml) in Raw264.7 cells. **(I)** The expression of SOCS3, p-ASK1, and ASK1 was measured in Raw264.7 cells after Axl knockdown. *n* = 3 per group; *p < 0.05, **p < 0.01, and ***p < 0.001 vs. control group; ^#^ *p* < 0.05, ^##^ *p* < 0.01, and ^###^ *p* < 0.001 vs. LPS group; ^$$^ *p* < 0.01 vs. LPS + PF group.

We further explored the mechanism by which PF induces SOCS3 expression. The Axl receptor is an upstream regulator of SOCS3 (van der Meer et al., 2014). As shown in Figures 6A,D, compared with the control group, PF significantly increased the phosphorylation of Axl in RAW264.7 and HUVEC. We further detected the expression of Axl ligand Gas6. As shown in Figures 6B,C,E, PF also significantly increased the level of Gas 6 protein and mRNA in RAW264.7 and HUVEC, which was consistent with the expression of *p*-Axl.

In addition, LPS was used to establish an inflammatory cell model. Compared with the LPS group, PF significantly increased the expression of Gas 6 and *p*-Axl in the LPS + PF group in Raw264.7 cells (Figures 6G,H). We then verified the importance of Axl by using its siRNA to measure the expression of SOCS3 and p-ASK1 in Raw264.7 cells. As shown in Figure 6I, PF markedly increased the expression of SOCS3 and decreased the phosphorylation of ASK1, which were eliminated by Axl knockout in Raw264.7 cells.

These data suggest that PF induces SOCS3 expression and inhibits ASK1 phosphorylation by activating the Axl receptor.

## Discussion

The major findings of this study are as follows: 1) radiation induces intestinal inflammation and slow blood flow in mice; 2) PF reduces mortality and improves RE in irradiated mice; 3) PF reduces radiation-induced intestinal injury and TF expression by decreasing ASK1 phosphorylation; 4) PF induces SOCS3 expression and inhibits the ASK1/TF axis *in vitro*; and 5) PF activates Gas6/Axl signaling to induce SOCS3 expression.

RE is a high-incidence complication in radiotherapy for patients with abdominal or pelvic cancer (Potten, 1990). Although the precision of chemotherapy has significantly improved with advancements in technology (Abbasoğlu et al., 2006), enteritis-related injuries caused by radiotherapy cannot currently be avoided and cured.

In this study, we first established the mouse RE model according to the study by Goldstein et al. (1976), with a total radiation dose of 14 Gy. We found that the mortality of mice exposed to radiation increased significantly over time (Figure 1A). Intestinal edema, infiltration of a large number of inflammatory cells, and severe intestinal structural damage were found in the colorectum after radiotherapy (Figure 1B), resulting in more severe weight loss in mice during the disease process (Figure 2B).

The effects of intestinal blood flow on RE have rarely been reported. Wang et al. (2004) found that the expression of TF was increased in the intestinal tract of rats that received radiotherapy and that hirudin and the platelet inhibitor clopidogrel (Driák et al., 2008) could alleviate RE by inhibiting platelet aggregation and reducing gastrointestinal damage. However, presently, there is no direct evidence supporting that poor intestinal blood circulation after radiotherapy is involved in RE. To the best of our knowledge, we were the first to use laser Doppler flowmetry to measure intestinal blood flow in mice. The results showed that the intestinal blood flow rate of mice decreased significantly on the sixth day after radiotherapy (Figure 2D) and that the ASK1/TF signaling pathway was significantly activated in the colon (Figure 3), indicating that local ischemia and hypoxia caused slow blood flow in the intestine.

In our previous study on the pathogenesis of chemotherapy-induced enteritis, we found that chemotherapy could cause microthrombi in the local circulation of intestinal blood vessels (Yu et al., 2020). In this study, there were many similarities between RE and chemotherapy-related enteritis, such as the observed significant reduction in intestinal blood flow after radiotherapy. It is noteworthy that the loss of a large number of intestinal collagen fibers within a short period of time completely comprised the intestinal function, with the severe inflammatory response leading to an increased vulnerability of the intestinal tract. In these conditions, not only there is aseptic inflammation induced by post-radiation apoptosis of vascular endothelial cells but also bacteriological inflammation induced by an impaired intestinal barrier resulting from the presence of bacteria in the blood, which brought further damage to the blood vessels; moreover, since the local ischemic microenvironment caused by clotting thrombi can further aggravate inflammation, it is reasonable to speculate that, in all enteritis models, RE is more likely to lethally compromise the intestinal tract, which is consistent with our expectation, and their mutual action jointly mediates the important mechanism of RE.

Currently, we are exploring alternatives to find potential treatments for RE in clinical practice. PF, a pinane monoterpene bitter glycoside, differs from red peony and white peony, a traditional Chinese medicine. This compound has analgesic, anti-inflammatory, antipyretic, and antispasmodic functions and has the advantage of presenting very low toxicity (He and Dai, 2011). At present, the effects of PF on the promotion of blood circulation and removal of blood stasis, along with its anti-inflammatory and anti-thrombotic effects, are its most relevant therapeutic properties (Jorch and Kubes, 2017; Fan et al., 2018). Our data showed that PF reduced mortality and improved weight loss in mice after radiotherapy in a dose-dependent manner (Figures 2A,B). The data from H&E staining and laser Doppler flowmetry indicated that PF could alleviate the inflammatory response and improve slow blood flow in mice after radiotherapy (Figures 2C,D). Indeed, PF has been shown to upregulate SOCS3 expression to reduce neuroinflammation and neuropathic pain by inhibiting ASK1 activity (Zhou et al., 2019). Here, we demonstrated that PF could induce SOCS3 expression and inhibit ASK1 phosphorylation (Figures 3C,D, 4A, 5A) *in vivo* and *in vitro*. More importantly, for the first time, we proposed a new mechanism of PF, involving the upregulation of SOCS3 expression, in which the upstream Gas6/Axl axis was activated by PF (Figure 6).

**FIGURE 7:**
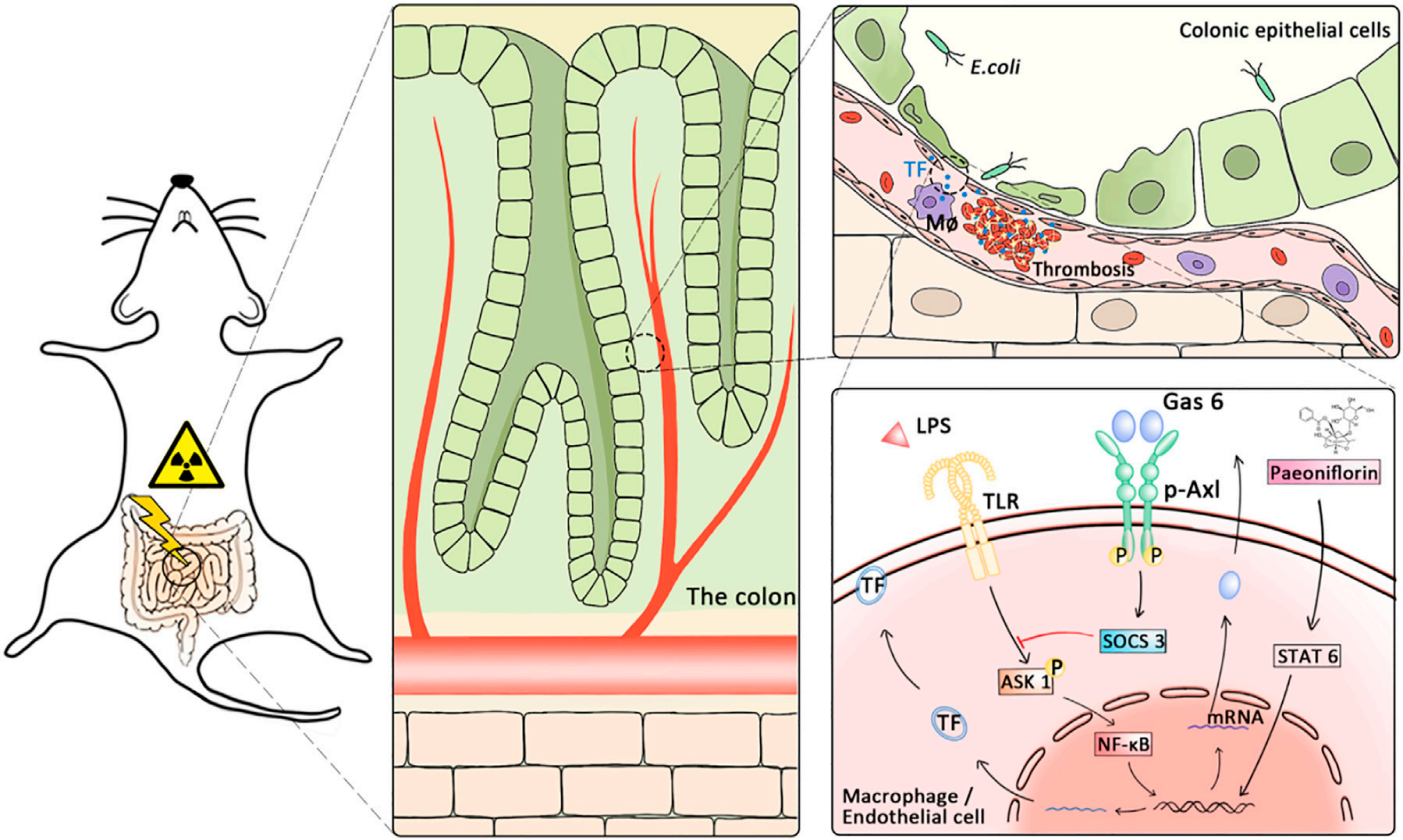
Proposed hypothesis on the mechanisms underlying the alleviating effects of PF on radiation enteritis. The TF released from vascular endothelial cells and macrophages after radiation caused thrombosis and microcirculation disorders. PF inhibits coagulation by promoting growth arrest-specific gene 6 release and *p*-Axl activation to upregulate SOCS3, subsequently reducing the expression of TF.

In conclusion, our findings suggest that slow blood flow caused by local microthrombi and inflammation in the intestine may be the core mechanism of RE. Furthermore, PF activated the Gas6/p-Axl/SOCS3 axis to alleviate RE by inhibiting p-ASK1 and TF expression (Figure 7).

## Data Availability

The raw data supporting the conclusion of this article will be made available by the authors, without undue reservation.
